# Supplementary material for: Fast boulder fracturing by thermal fatigue detected on stony asteroids
Source: Nat Commun. 2024 Jul 30;15:6206. doi: 10.1038/s41467-024-50145-y (PMC11289370; doi:10.1038/s41467-024-50145-y)
Supplement: Supplementary file 1 — Supplementary Information [file 41467_2024_50145_MOESM1_ESM.pdf]

Supplementary Information

Fast boulder fracturing by thermal fatigue detected on stony asteroids

A. Lucchetti[1*], S. Cambioni[2], R. Nakano[3,7], O.S. Barnouin[4], M. Pajola[1], L. Penasa[1], F. Tusberti[1], , K.T. Ramesh[5], E. Dotto[6], C.M. Ernst[4], R.T. Daly[4], E. Mazzotta Epifani[6], M. Hirabayashi[3,7], L. Parro[8,9,10], G. Poggiali[11,12], A. Campo Bagatin[8,13], R.-L. Ballouz[4], N. L. Chabot[4], P. Michel[14,15], N. Murdoch[16], J.B. Vincent[17], Ö. Karatekin[18], A.S. Rivkin[4], J.M. Sunshine[19], T. Kohout[20], J.D.P. Deshapriya[6], P.H.A. Hasselmann[6], S. Ieva[6], J. Beccarelli[1], S.L. Ivanovski[21], A. Rossi[22], F. Ferrari[23], C. Rossi[1], S. D. Raducan[24], J. Steckloff[25], S. Schwartz[25], J.R. Brucato[11], M. Dall'Ora[26], A. Zinzi[27], A. F. Cheng[4], M. Amoroso[28], I. Bertini[29], A. Capannolo[16], S. Caporali[11], M. Ceresoli[23], G. Cremonese[1], V. Della Corte[26], I. Gai[30], L. Gomez Casajus[30], E. Gramigna[30], G. Impresario[28], R. Lasagni Manghi[30], M. Lavagna[23], M. Lombardo[30], D. Modenini[30], P. Palumbo[31], D. Perna[6], S. Pirrotta[28], P. Tortora[30], M. Zannoni[30], G. Zanotti[23].

# Boulder	Boulder Diameter (m)	# Fractures	Fracture Length (m)	Fracture length / Boulder Diameter
1	2.10	1	1.52	0.72
2	2.39	1	1.41	0.59
3	3.95	1	0.80	0.20
4	3.73	3	0.73 -1.35	0.2 - 0.36
5	1.07	1	0.97	0.90
6	4.44	3	0.99 - 1.37	0.22 - 0.31
7	1.84	2	0.5 - 0.69	0.27 - 0.37
8	1.69	1	0.69	0.41
9	2.18	1	0.43	0.20
10	4.77	2	0.75 - 1.67	0.15 - 0.35
11	3.51	2	1.02 - 1.67	0.29 - 0.47
12	2.52	2	0.68 - 0.84	0.27 -0.33
13	3.69	3	1.01 - 1.88	0.27 - 0.51
14	5.10	2	0.78 - 0.87	0.15 - 0.17
15	2.88	2	0.78 - 1.1	0.27 - 0.38
16	3.34	2	1.01 - 1.19	0.30 - 0.36

17	3.41	1	0.91	0.27
18	6.62	6	0.82 - 3.14	0.12 - 0.47
19	2.35	1	0.79	0.34
20	4.46	2	1.01 - 1.23	0.23 - 0.28
21	2.78	1	1.34	0.48
22	4.01	3	0.6 - 0.84	0.15 - 0.21
23	3.08	1	1.03	0.33
24	4.70	2	1.63 - 1.69	0.35 - 0.36
25	5.29	1	2.50	0.47
26	3.88	2	1.85 - 2.0	0.48 - 0.52
27	3.06	1	2.23	0.73
28	3.44	1	1.24	0.36
29	1.86	1	0.52	0.28
30	3.12	1	0.78	0.25

Supplementary Table 1. Fracture length and hosting boulder diameter. We report the diameter (in meters) of each boulder hosting fractures, which is defined as the value of each ellipse's major axis fitted to each boulder, along with the number of mapped fractures. In addition, the range of both the length (in meters) and the ratio between the length of fractures and the hosting boulder dimension are reported. Such findings reveal that all fractures are smaller than the dimension of the hosting boulder. The gray rows represent the three boulders that have been thermophysically modeled.

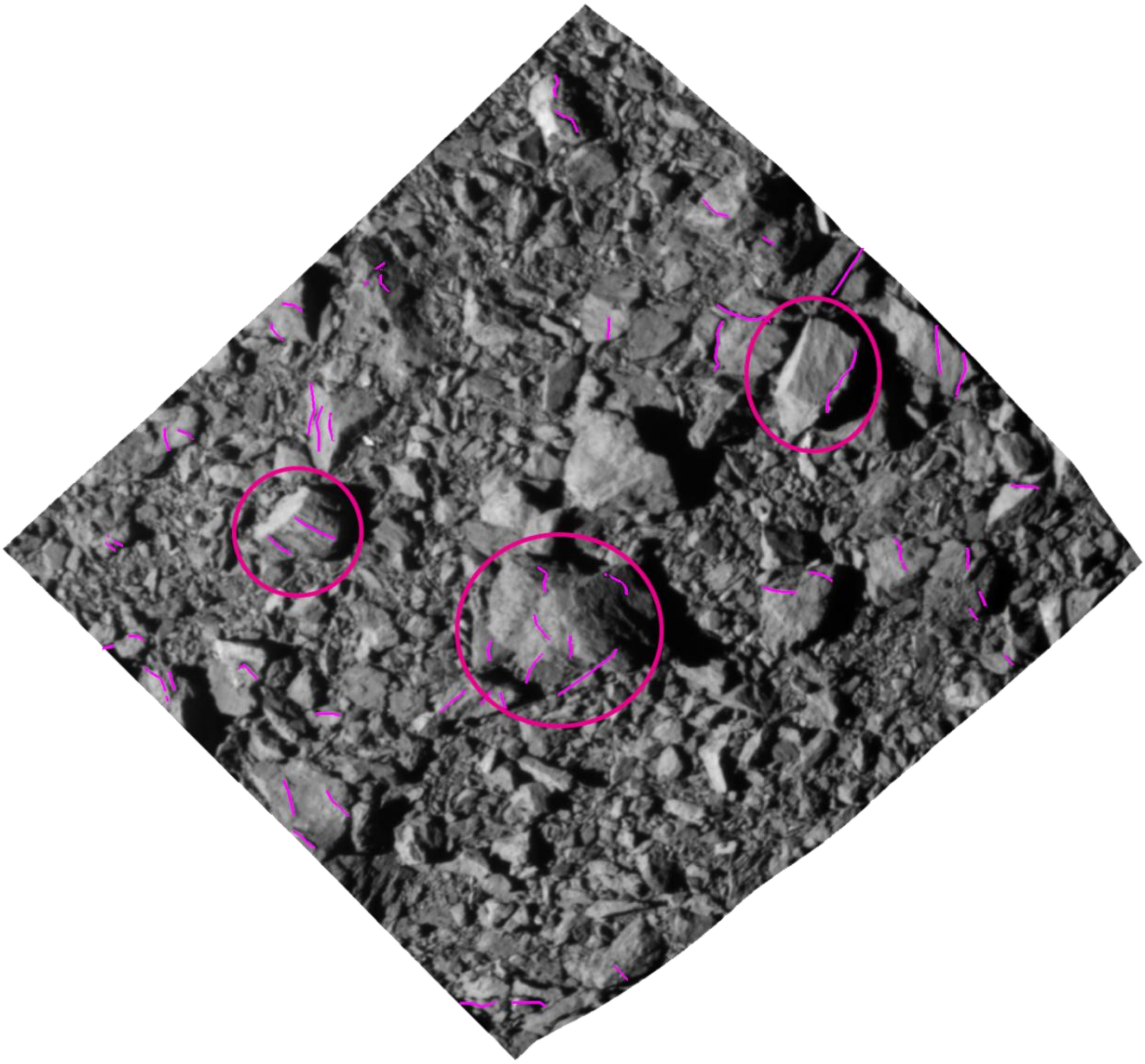

Supplementary Figure 1. The three boulders where the thermophysical modeling has been applied: Atabaque Saxum (center, 6.62 m across and characterized by 6 cracks), a boulder with 1 crack (right, 5.29 m across), and a boulder with 2 cracks (left, 3.51 m in diameter).

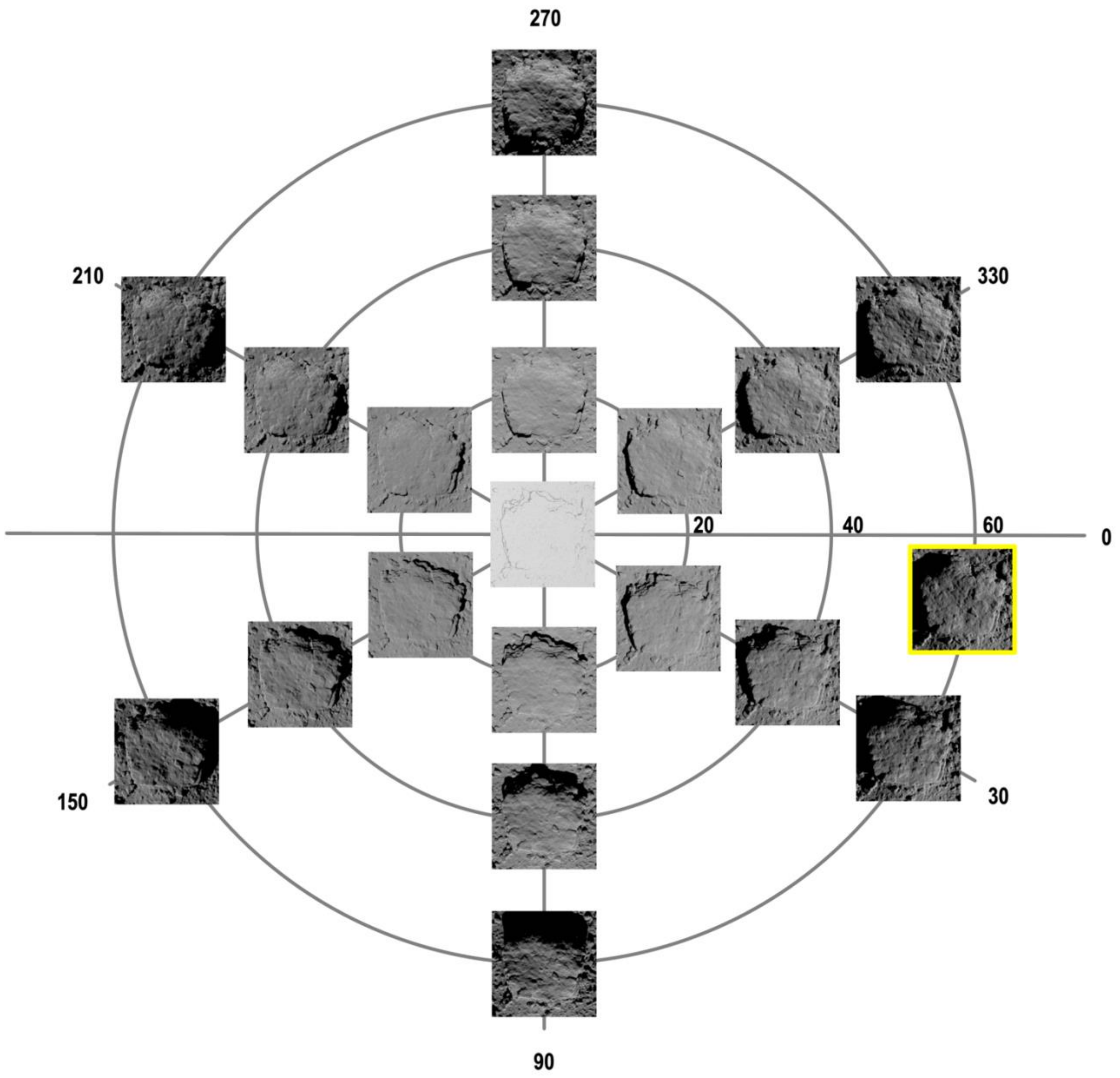

Supplementary Figure 2. The different illumination conditions used to understand how different lighting may play a role when identifying crack orientations. We decided to choose a boulder of Bennu because this one is affected by lineaments oriented in all directions. Moreover, its digital terrain model (DTM) has been produced by the OSIRIS-REX Laser Altimeter (Daly et al., 2017) with a 5 cm ground sampling distance.

Statistical analysis on the validity of fractures' orientation
We here report the analyses we have accomplished to strengthen the validity of our findings (preferred orientation of boulder's fractures). In particular, we investigate:
1) the probability that the observed azimuthal distribution arose by chance from a uniform Distribution, i.e. from randomly oriented fractures;
2) the sampling bias effect, implying that fractures with azimuth similar to the sunlight direction are less likely to be identified, and thus are not present from the dataset.

The observed azimuthal distribution coming from the fractures analysis performed in this work, coupled with a histogram representation is reported in Supplementary Figure 3. The resulting azimuth

mean value and standard deviation, together with and their relative bootstrapped CI (95% confidence interval) are presented:

- Mean: 150.04°, 95% CI: -15.89, 16.75
- Std: 41.56 , 95% CI: -6.92, 12.79

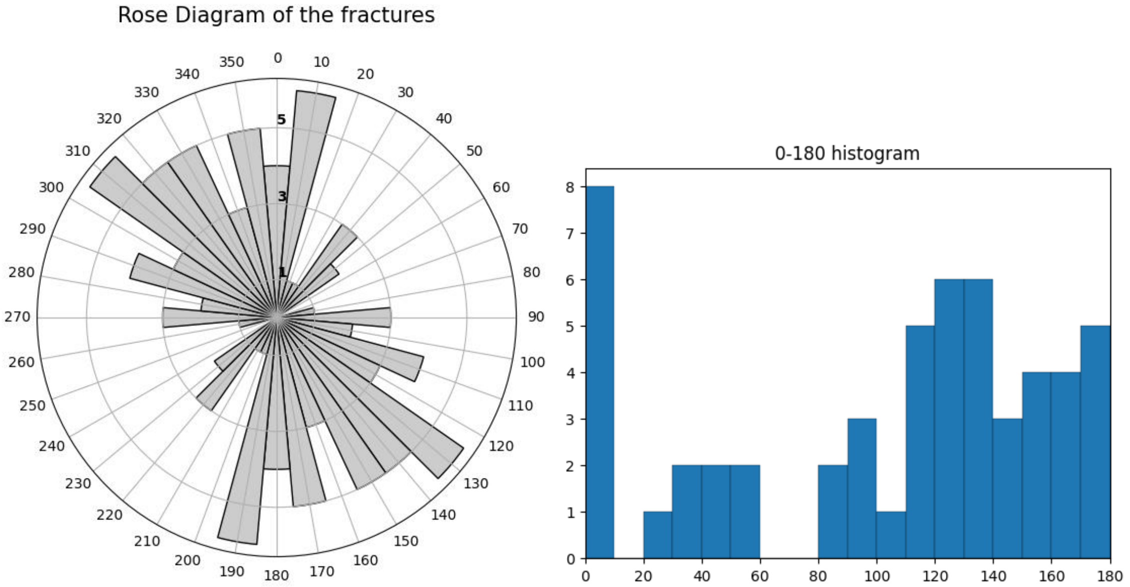

Supplementary Figure 3. Left: Rose diagram of fractures analyzed in this work. Right: histogram representation of such fractures.

We investigate how likely it is to generate such a distribution as function of angular values by chance with statistical properties (mean and std) similar to our dataset. A clean null hypothesis for this study would result in a purely random orientation of fractures (with a process that could produce such a uniform distribution). If this hypothesis is verified, then any clustering in our data would arise by chance. We can establish a null hypothesis when the observed distribution arises from the sampling of a uniform distribution. To reject the null hypothesis, we need to demonstrate that our distribution, or one that is very similar to ours, cannot be generated by chance. Hence, we sample multiple times a random uniform distribution with the same sample size of the collected data.

To generate a uniform distribution, we first produce an example with the same number of samples as in our dataset, using a uniform distribution from 0° to 180°. The first plot of Supplementary Figure 4 shows our observed distribution as a reference. Then, we can repeat this sampling for a large number of cases (100000) – some of which are shown below (Supplementary Figure 4) -- and demonstrate that the likelihood to produce a standard deviation similar to the one we derived is small, i.e. near 0.129 %. This is of course limited from a statistical point of view, but it provides the indication that is quite unlikely that our dataset arises by chance from a uniform distribution (this can be defined with > 99 % confidence).

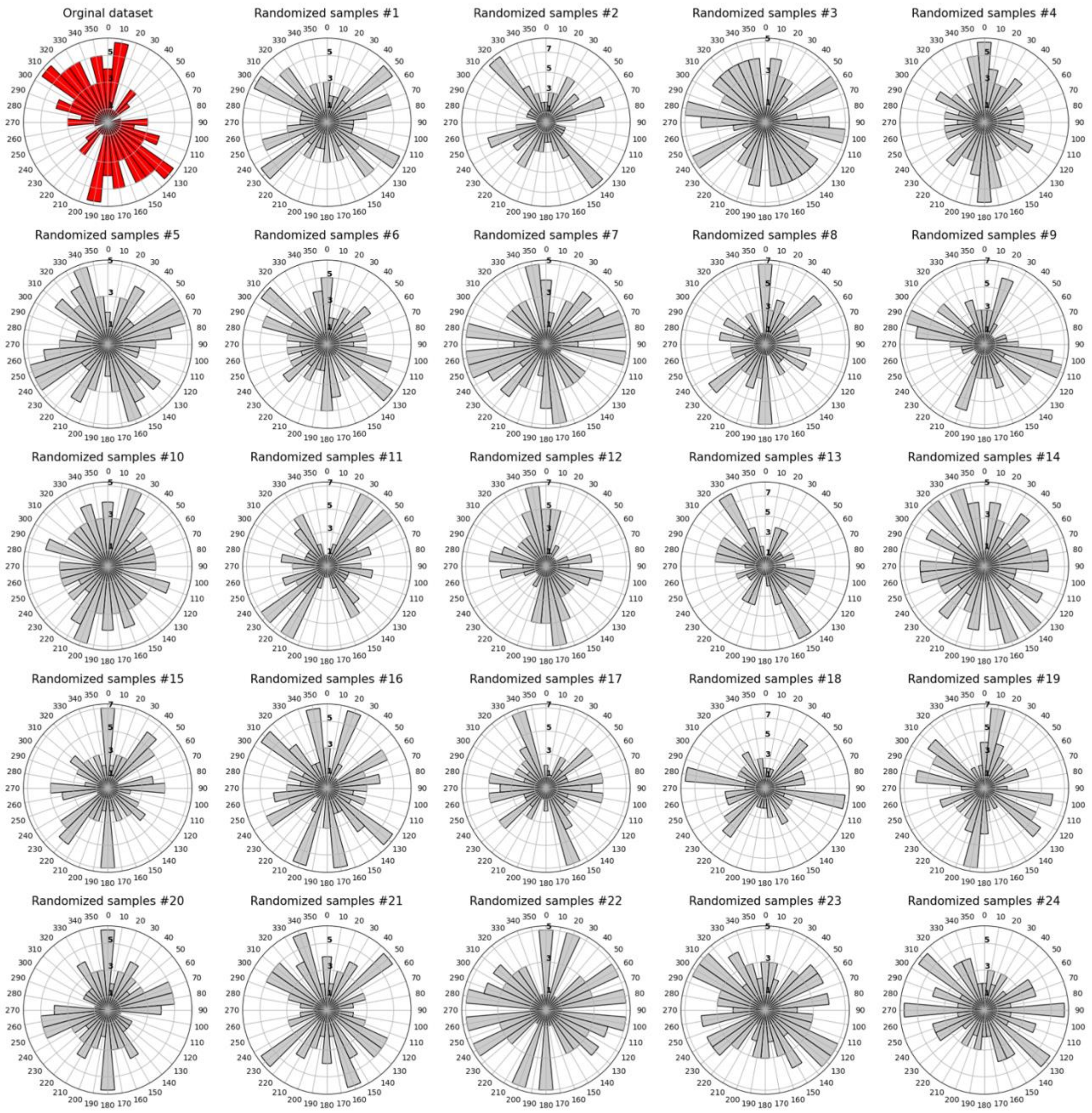

Supplementary Figure 4: Original dataset (in red) as a reference and some examples of generated uniform distributions.

Afterwards, we can make a simplified model of the expected effect of the lighting bias. The idea is that for fractures with the same azimuth of the Sunlight direction, the detection is impossible or at least very difficult.

To model this condition, we can start with a uniform distribution, where fractures with an azimuthal orientation similar to the one of the incoming light are more likely to remain undetectable, hence are not inside the final dataset. We report below an example that should clarify the issue, even if we are aware that such analysis is quite limited from a statistical perspective, but still quite illustrative.

The implementation of the model looks like as follows:

```
def random_dataset(N, loc=90, std=10, corrective_factor=1):
nprob = norm(loc, std)
```

```
numbers = []
while len(numbers) != N:
value = np.random.uniform(0,180, 1)[0]
p = nprob.pdf(value)/nprob.pdf(loc) * corrective_factor
reject = np.random.choice([True, False], p=[p, 1-p])
if not reject:
numbers.append(value)
return np.array(numbers)
```

The sun illuminates the scene with an azimuth angle that we assume equal to 100°. In particular, after considering that the subsolar point is located -10°N, 60°E with respect to the location of the boulders, the sun azimuth value is 101.51°. Below, we report some plots to have a clear idea of the behavior of the abovementioned simplified model (Supplementary Figure 5).

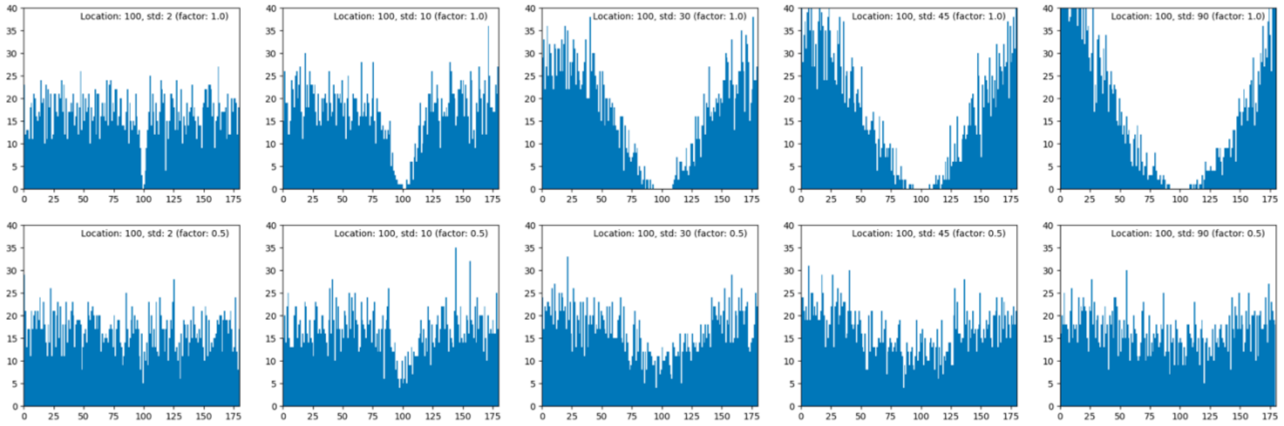

Supplementary Figure 5: Output of the simplified model including the expected effect of the lighting bias.

Afterwards, we run again the simulation we did above by repeating the sampling of such distribution, with the same number of samples of the original dataset. As standard deviation (std) of the gaussian we use a value of 22. As shown in the next plot (Supplementary Figure 6), it is clear that this std value generates a synthetic dataset with the same standard deviation as the one observed in real data. We highlight that change of such a value will end up with standard deviation values that are less realistic, hence making difficult to reproduce a dataset with statistical properties similar to our original one.

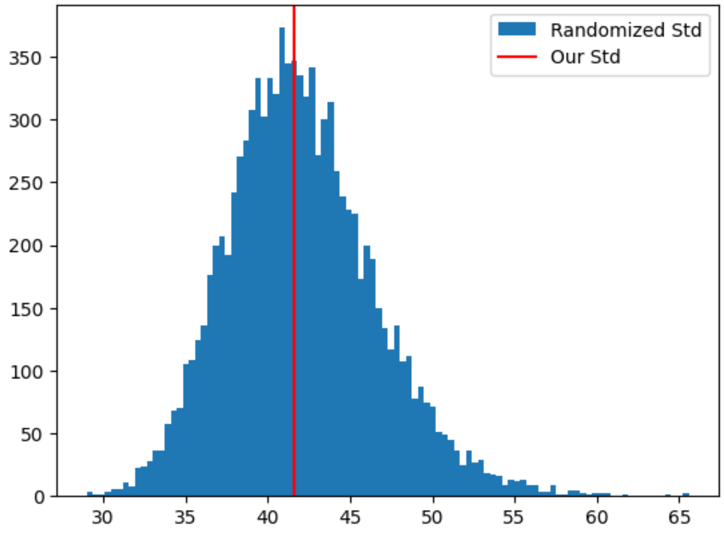

Supplementary Figure 6 Synthetic dataset with the same std as the one observed in original data.

Such findings suggest that a random process, as the one modelled above, can generate a dataset with a similar std as the original one. Nevertheless, the process has the effect of removing samples around the 100° azimuth, clustering the values in the opposite direction (10°) (Supplementary Figure 7a). Hence, in terms of average azimuth value, the generated datasets are quite different. In order to help the reader, we rearrange the dataset to be wrapped around a 90° angle, rather than 180°, hence making the dataset more obviously a gaussian (Supplementary Figure 7b).

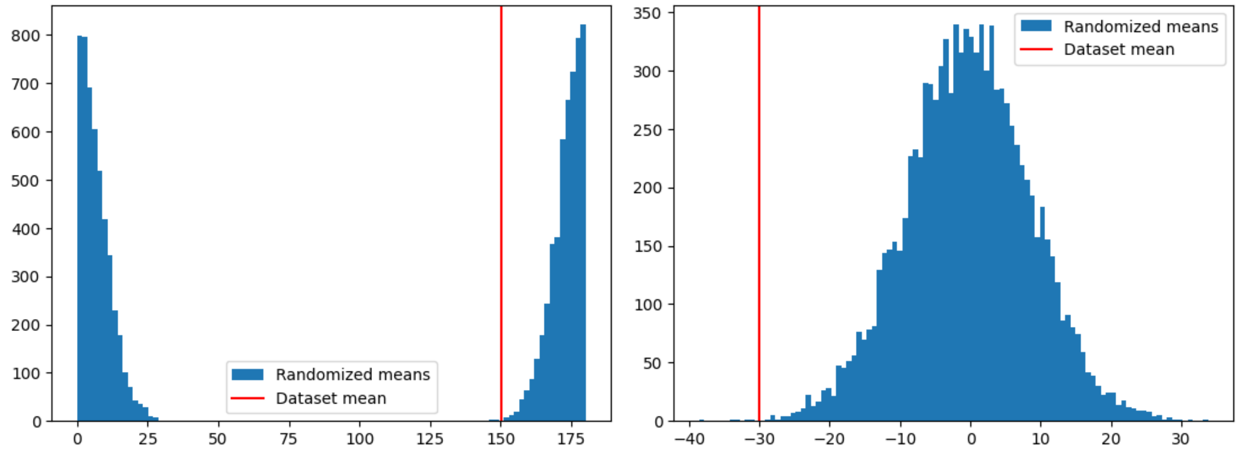

Supplementary Figure 7a and 7b: Synthetic dataset including the illumination bias effect.

This plot demonstrates that is quite unlikely to generate the observed average orientation of fractures by a uniform process including the illumination bias effect. Specifically, 0.05 times out of 100, we obtain a mean similar to the one of our original dataset. The plot highlights that it is quite difficult to get a mean of -30 from a randomized sample plus the illumination bias. Furthermore, it shows that the illumination bias would create a decrease of observations at azimuth corresponding to the sun azimuth, hence altering the dataset, and moving the average value to the azimuth located at sun azimuth of + 90°.

The above analyses support the fact that i) the dataset is unlikely to be simply the result of a uniform distribution, hence leading to the rejection of the null hypothesis (the dataset comes from a uniform distribution), and ii) the dataset is unlikely to result from a uniform distribution altered by the illumination bias alone. Indeed, the direction where the azimuth values are clustered differs from the one that would be expected with an illumination bias with azimuth ~100°.

We highlight that from a statistically perspective, such a result does not prove that the dataset cannot be derived from the superimposition of a uniform distribution process and additional effect, rather that our original dataset does not directly come from a uniform distribution, neither from a uniform distribution plus the illumination bias.

Other biases (e.g., boulders' shape) should also probably be considered, but the limited dataset and the lack of new data (until HERA mission, whose arrival will be in Dec 2026) make a more advanced analysis quite difficult and limited.
